# A Robust Approach to Early Glaucoma Identification from Retinal Fundus Images using Dirichlet-based Weighted Average Ensemble and Bayesian Optimization

**DOI:** 10.2174/0115734056335762250128095107

**Published:** 2025-02-27

**Authors:** Mohamed Mouhafid, Yatong Zhou, Chunyan Shan, Zhitao Xiao

**Affiliations:** 1 School of Electronics and Information Engineering, Hebei University of Technology, Tianjin 300401, China; 2 NHC Key Laboratory of Hormones and Development, Tianjin Key Laboratory of Metabolic Diseases, Chu Hsien-I Memorial Hospital & Tianjin Institute of Endocrinology, Tianjin Medical University, Tianjin 300134, China; 3 School of Life Sciences, Tiangong University, Tianjin 300387, China

**Keywords:** Ensemble Learning, Transfer learning, Image classification, Glaucoma detection, CNN, Bayesian Optimization

## Abstract

**Objective:**

Glaucoma is a leading cause of irreversible visual impairment and blindness worldwide, primarily linked to increased intraocular pressure (IOP). Early detection is essential to prevent further visual impairment, yet the manual diagnosis of retinal fundus images (RFIs) is both time-consuming and inefficient. Although automated methods for glaucoma detection (GD) exist, they often rely on individual models with manually optimized hyperparameters. This study aims to address these limitations by proposing an ensemble-based approach that integrates multiple deep learning (DL) models with automated hyperparameter optimization, with the goal of improving diagnostic accuracy and enhancing model generalization for practical clinical applications.

**Materials and Methods:**

The RFIs used in this study were sourced from two publicly available datasets (ACRIMA and ORIGA), consisting of a total of 1,355 images for GD. Our method combines a custom Multi-branch convolutional neural network (CNN), pretrained MobileNet, and DenseNet201 to extract complementary features from RFIs. Moreover, to optimize model performance, we apply Bayesian Optimization (BO) for automated hyperparameter tuning, eliminating the need for manual adjustments. The predictions from these models are then combined using a Dirichlet-based Weighted Average Ensemble (Dirichlet-WAE), which adaptively adjusts the weight of each model based on its performance.

**Results:**

The proposed ensemble model demonstrated state-of-the-art performance, achieving an accuracy (ACC) of 95.09%, precision (PREC) of 95.51%, sensitivity (SEN) of 94.55%, an F1-score (F1) of 94.94%, and an area under the curve (AUC) of 0.9854. The innovative Dirichlet-based WAE substantially reduced the false positive rate, enhancing diagnostic reliability for GD. When compared to individual models, the ensemble method consistently outperformed across all evaluation metrics, underscoring its robustness and potential scalability for clinical applications.

**Conclusion:**

The integration of ensemble learning (EL) and advanced optimization techniques significantly improved the ACC of GD in RFIs. The enhanced WAE method proved to be a critical factor in achieving well-balanced and highly accurate diagnostic performance, underscoring the importance of EL in medical diagnosis.

## 1 INTRODUCTION

Glaucoma is a chronic optic neuropathy condition characterized by a gradual and slow destruction of the optic nerve fiber layer. It results in irreversible blindness in the absence of proper treatment [1]. The diagnosis of glaucoma typically includes the use of RFIs, which are very common in clinical practice. The retina of the eye provides a convenient means to observe how the body maintains balance. It allows for the observation of vascular, neural, and connective tissues without causing harm. Systemic diseases may show signs in the fundus that help us find, diagnose, stage, keep an eye on, and treat the disease. Retinal photography is readily accessible and can be performed by medical personnel with minimal training. Furthermore, capturing the RFIs of a patient can be done within a few minutes. The patient can promptly be referred to a specialist if any issues are detected [2]. Previous research in ophthalmology has demonstrated that RFIs serve as effective diagnostic tools for conditions such as macular degeneration, refractive errors, and glaucoma. Additionally, RFIs can be employed to assess systemic risks associated with cardiovascular diseases [3]. This evidence suggests that retinal photography holds promise for broad applications in disease screening programs. The RFI enables the visual distinction of the optic disc (OD) into two discernible regions: the optic cup, positioned centrally and appearing bright, and the neuro-retinal rim, forming the peripheral portion. While both the optic cup and disc are present in all individuals, glaucomatous eyes typically exhibit an abnormally enlarged optic cup relative to the OD, which serves as a distinguishing characteristic. Although RFIs are widely available and hold significant diagnostic value, the manual assessment of these images for GD is inherently time-consuming, subjective, and susceptible to variability among clinical experts [4]. This limitation poses a significant challenge to the timely diagnosis and management of the disease, particularly in large-scale screening initiatives.

Automated approaches have the potential to contribute to cost reduction and the facilitation of large-scale screening programs by delivering accurate predictions in a prompt manner [5]. The use of machine learning (ML) algorithms has gained traction in automating GD processes. They serve as an umbrella term for advancements in computer systems. Moreover, they are designed to execute a range of tasks related to artificial intelligence (AI), including diagnostics, robot control, identification, planning, forecasting, and more. This progress can involve either refining existing systems or creating entirely new ones from scratch. ML encompasses a subfield known as DL, which focuses on Artificial Neural Networks. These algorithms are inspired by the structure and functioning of the brain [6] and are capable of processing both structured and unstructured data. In contrast to supervised ML, which relies on human-defined features, DL independently discovers and refines features, enabling the extraction of complex, high-level patterns with minimal manual effort. DL offers an effective approach for addressing complex, highly nonlinear problems.

A fundamental architecture in DL is the CNN, which excels in image-related tasks due to its ability to recognize spatial hierarchies within images. CNNs consist of layers that automatically identify low-level features, such as edges and textures, in the initial layers and progressively build more complex representations in deeper layers [7, 8]. This hierarchical approach to feature extraction makes CNNs highly effective in detecting subtle patterns, such as changes in the OD associated with glaucoma, in medical images. However, training CNNs for specialized tasks, particularly in medical imaging, requires careful attention to both the network architecture and the selection of hyperparameters.

Key factors affecting the performance of CNNs in medical imaging tasks, including GD, include hyperparameters like learning rate, filter dimensions, and network depth [9]. Manually tuning these hyperparameters can be time-consuming and computationally demanding [10]. Conventional methods such as grid search and random search explore fixed sets of hyperparameters, identifying configurations that optimize performance [11]. However, these methods can be inefficient, especially when dealing with complex models and large search spaces. BO offers a more advanced approach to address this challenge [12]. BO is a probabilistic model-based optimization technique that uses a surrogate model, typically a Gaussian process, to approximate the objective function and guide the selection of hyperparameters based on previous evaluations. This strategy allows for a more efficient exploration of the search space, minimizing the number of evaluations needed to identify the optimal hyperparameter configuration. BO is particularly advantageous when hyperparameter evaluations are costly, such as when training DL models on large datasets [13].

While optimizing individual models is crucial, EL methods offer a means to enhance performance by combining the predictions of multiple models [14]. These methods can reduce overfitting and improve generalization by leveraging the diversity of different models. Common strategies in EL include bagging, boosting, and stacking, which integrate models in various ways to increase robustness and minimize errors [15, 16]. One notable method is WAE, which combines predictions from multiple models by assigning weights based on their performance. This approach enables the ensemble to capitalize on the strengths of each model while mitigating their weaknesses.

However, traditional WAE methods can be limited by how weights are assigned. In conventional WAE, weights are typically based on performance metrics (*e.g*., ACC, PREC, SEN). While this can offer some improvement over individual models, it may not always lead to an optimal distribution of contributions, especially when models perform differently across various parts of the dataset. Consequently, some models may dominate the decision-making process, reducing the overall performance of the ensemble.

Motivated by these challenges, this study proposes a Dirichlet-based WAE method. This approach uses the Dirichlet distribution to assign optimal weights to each model, ensuring a more balanced contribution from all ensemble members. The Dirichlet distribution, a probability distribution often used to model uncertain or proportional outcomes, allows for more effective management of individual model contributions. This ensures that the ensemble remains robust, with no single model disproportionately influencing the final decision. Additionally, by addressing the limitations of existing approaches, this work seeks to improve diagnostic outcomes and facilitate large-scale screening programs. This paper introduces several novel contributions designed to enhance the performance of automated GD through the use of DL and ensemble methods:

• A custom-designed Multi-branch CNN architecture is proposed to enhance feature extraction from RFIs. This model incorporates dual branches with specialized convolutional pathways that independently capture distinct spatial features, resulting in a more comprehensive representation of glaucoma-specific patterns.

• The classification components of the MobileNet, DenseNet201, and Multi-branch CNN models are automatically designed using BO. This approach improved model performance by better adapting the network to the specific requirements of GD.

• A Dirichlet-based WAE method is proposed, which integrates the optimized CNN models from the BO phase into a unified ensemble. The WAE method utilizes the Dirichlet distribution to assign optimal weights to each model, ensuring a balanced contribution from each member of the ensemble.

• A selective fine-tuning strategy is applied to the MobileNet and DenseNet201 architectures to further enhance the ensemble's performance. This approach adjusts parameters in specific layers to capture relevant features for the task while preserving essential pre-trained knowledge, resulting in a validation ACC of 97.04% and a test ACC of 95.09%, surpassing the performance of existing methods.

The rest of this paper is organized as follows: Section 2 reviews recent methodologies for GD using RFIs. Section 3 details the materials and methods, including data collection and pre-processing procedures, a description of the three proposed base-learners, an overview of the BO stage, an explanation of the Dirichlet-based WAE strategy, a description of the fine-tuning process, and an outline of the performance evaluation metrics and experimental setup. Section 4 presents the experimental results. Section 5 provides a discussion of the findings. Finally, Section 6 concludes the study.

## 2 RELATED WORKS

In recent years, numerous automated approaches have been developed to facilitate GD through ML and DL methodologies. These techniques aim to enhance diagnostic PREC, improve interpretability, and minimize manual efforts, thus supporting ophthalmologists in clinical practice. This section presents an analysis of predominant ML-based, DL-based, and EL-based methods within the literature for GD, examining the strengths and limitations associated with each approach.

Traditional ML techniques have been extensively employed in the analysis of RFIs by leveraging handcrafted features that encapsulate attributes pertinent to glaucoma diagnosis. These approaches often depend on dimensionality reduction and classification algorithms, necessitating substantial pre-processing and feature engineering efforts. Although generally less computationally demanding than DL techniques, their effectiveness is contingent upon the quality and relevance of the extracted features. Agrawal *et al.* [17] introduced an automated GD method employing quasi-bivariate variational mode decomposition (QB-VMD) on RFIs. The QB-VMD technique decomposes images into several intrinsic components, from which features are extracted. The ReliefF algorithm was subsequently applied to identify the most significant features, selecting those that best distinguish between classes. Dimensionality reduction of the chosen features was achieved using singular value decomposition (SVD), optimizing input for classification by an SVM. This method was validated on the RIM-ONE dataset, comprising 505 images, and achieved ACC scores of 85.94% and 86.13% through three-fold and ten-fold cross-validation, respectively. Kirar *et al.* [18] developed a hybrid approach for GD, integrating discrete wavelet transform (DWT) and empirical wavelet transform (EWT) to extract a comprehensive set of features from RFIs. The combination of DWT, which captures multi-resolution features, with EWT, which adapts to localized image characteristics, enhances SEN to subtle distinctions between glaucomatous and non-glaucomatous images. The extracted features were subsequently fused to create a holistic representation of the retinal structure. Singular value decomposition (SVD) was used to reduce feature dimensionality, retaining the most critical information and eliminating redundancies, with classification then performed using SVM. This method was assessed via ten-fold cross-validation, yielding an ACC of 83.57%. Maheshwari *et al.* [19] proposed a method utilizing variational mode decomposition (VMD) for image disentanglement, followed by ReliefF feature selection and classification through the least squares support vector machine (LS-SVM). The VMD process decomposed RFIs into distinct intrinsic components, facilitating the extraction of more relevant and discriminative features. ReliefF was applied to refine the feature set, thereby enhancing the model's ACC by filtering out irrelevant features. LS-SVM, known for its efficiency in high-dimensional classification tasks, was employed for the final classification. This hybrid model achieved an ACC of 95.19% through three-fold cross-validation on a local retinal dataset.

In contrast to traditional ML techniques, DL (particularly through CNNs) has made substantial strides in automating feature extraction and facilitating end-to-end learning. CNN-based models, trained directly on data, enable the network to autonomously learn features relevant to glaucoma, obviating the need for manual feature engineering and yielding superior performance when ample data is available. Bajwa *et al.* [20] introduced a multi-phase strategy for OD identification and GD using RFIs. Initially, OD localization is achieved using a Regions-with-CNN (RCNN) model, which the authors validated on multiple publicly available datasets. In the subsequent phase, a CNN architecture is applied for glaucoma classification, attaining an AUC of 0.874 on the ORIGA dataset. D’Souza *et al.* [21] proposed AlterNet-K, a parameter-efficient model based on an alternating architecture that integrates ResNets with multi-head self-attention (MSA) to enhance model generalizability by leveraging the strengths of both structures. This design consists of an initial convolutional layer followed by five intermediate stages, each incorporating a ResNet block, an MSA block, and a max-pooling layer. Additionally, by limiting the maximum channel length in each stage, the model effectively reduces parameter count, with “K” in AlterNet-K signifying this maximum. In contrast to traditional ResNet models, AlterNet-K omits residual bottleneck blocks, producing final feature maps without max pooling. The model was trained on the Rotterdam EyePACS AIROGS dataset, which includes 113,893 RFIs from 60,357 subjects, achieving a classification ACC of 91.6%. Juneja *et al.* [22] developed CoG-NET, an adaptation of the Xception architecture with 85 layers, specifically for GD. The network integrates convolutional and max-pooling layers with batch normalization (BN) and dropout for improved stability and regularization. The 3D output is transformed into a 2D feature map via global max pooling, and a fully connected layer with sigmoid activation performs the final classification. CoG-NET was optimized with the Adadelta optimizer to distinguish between normal and glaucomatous images, achieving 93.5% ACC on a dataset of 2,172 images aggregated from four sources. Liao *et al.* [23] presented EAMNet, an interpretable DL model designed specifically for GD. EAMNet utilizes a ResBlock-based framework, incorporating multiple convolutional and pooling layers to extract features from RFIs. Multi-layer average pooling is employed to capture multi-scale features, bridging the gap between semantic information and localization. Central to EAMNet’s interpretability is the evidence activation mapping (EAM) mechanism, which emphasizes glaucoma-specific regions within images by using a weighted sum of feature maps, thus highlighting discriminative regions. The model is configured with a low filter count to minimize redundancy and includes dropout and BN to mitigate overfitting. EAMNet achieved an AUC of 0.88 on the ORIGA dataset, demonstrating both ACC and interpretability in clinical contexts.

Transfer Learning (TL) has emerged as a prominent technique in GD, utilizing pre-trained CNN models to decrease training time and improve ACC, particularly when working with limited data. These models, initially trained on large image datasets, are fine-tuned to adapt to the specific task of GD within RFIs. Leonardo *et al.* [24] introduced a novel framework aimed at enhancing the quality of RFIs captured by fundus cameras, employing DL generative models to upgrade lower-quality samples. A critical component of this pipeline involves assessing whether the image quality was improved, maintained, or degraded. The authors developed a quality evaluation model, RetinaQual-Evaluator, based on the lightweight EfficientNetB0 architecture to classify RFIs by quality level. Additionally, using a dataset of 3,187 images from seven sources, the framework achieved a classification ACC of 93.1%. Almansour *et al.* [25] proposed a multi-stage framework for GD, beginning with OD extraction using an R-CNN and followed by classification with VGG16. The mask R-CNN architecture comprises a backbone for feature extraction, a region proposal network, and three head branches for classification, detection, and instance segmentation. However, only the detection head was utilized for region-of-interest (ROI) localization. In the classification phase, the VGG16 backbone with pre-trained weights was augmented with two additional fully connected layers, while the final classification layer was modified for binary classification, incorporating a softmax activation function. Trained on a dataset of 3,771 images from seven sources, this model achieved an ACC of 78%. Martins *et al.* [26] developed an interpretable model for glaucoma diagnosis capable of offline deployment on mobile devices. Their approach employs MobileNetV2 as the backbone for feature extraction, taking advantage of its efficiency in mobile applications. The extracted features undergo global average pooling (GAP) to flatten the feature maps, which are then passed through two fully connected layers with substantial dropout to mitigate overfitting. Evaluated on 3,231 images from seven distinct datasets, the model attained an ACC of 87%, demonstrating both ACC and suitability for mobile-based diagnosis.

EL, which integrates multiple models to enhance predictive ACC and resilience, has shown potential in GD by capturing a wide range of features from diverse CNN architectures. Cho *et al.* [27] introduced an ensemble strategy that combines 56 CNN models to improve glaucoma grading ACC in RFIs. This ensemble was created by using 2 color channels of RFIs, applying seven different image filters, and employing four CNN architectures, resulting in a total of 56 unique CNN models. The final decision was made by averaging the probabilities generated by each model. Their dataset consisted of 3,460 RFIs from 2,204 subjects, with the ensemble method achieving an ACC of 88.1%. Serte *et al.* [28] developed an ensemble CNN method for GD that begins by calculating saliency in RFIs, followed by thresholding to identify and map the most prominent regions. This saliency map guides the ensemble approach, focusing on key areas such as the OD. The method combines three CNN architectures (AlexNet, ResNet-50, and ResNet-152) with graph-based saliency to highlight changes in image intensity, contrast, and patterns, which are essential for detecting both early and advanced glaucoma. Using the Harvard dataset with 1,542 images, the ensemble achieved 88% ACC.

Despite these advancements, several challenges persist in automated GD. Traditional ML techniques, though effective with small datasets, depend heavily on handcrafted features, which often fail to capture the subtle, complex characteristics of glaucoma in RFIs, resulting in limited ACC despite advanced feature selection and dimensionality reduction methods. In DL, particularly with TL, many studies apply pre-trained CNN models with limited fine-tuning, potentially missing glaucoma-specific features. Additionally, while ensemble approaches improve robustness by integrating multiple models, the reported ACC scores remain around 88%, indicating room for further improvement. Another limitation across these studies is the absence of automated hyperparameter optimization, as most models depend on manually selected parameters. This manual selection process may hinder performance and limit the adaptability of these models to new datasets or varying imaging conditions. This study addresses key gaps in the field by introducing a robust medical recognition system that leverages fine-tuning, EL, and BO. This approach not only advances current methodologies for GD but also provides a comprehensive framework that can inform and guide future research efforts in medical image analysis.

## 3 MATERIALS AND METHODS

This section presents a detailed account of the methodologies employed to tackle the task of GD from RFIs. It begins with an explanation of the data collection and pre-processing steps, highlighting the creation of a combined dataset, OD extraction, and image normalization procedures. The backbone architectures are then discussed, including the proposed Multi-branch CNN and the modifications introduced to MobileNet and DenseNet201. Furthermore, the application of BO for fine-tuning hyperparameters across all models is described. Finally, a Dirichlet-based WAE strategy is proposed to leverage the complementary strengths of individual models, enhancing overall performance. Each of these methodologies is explored in detail in the subsequent subsections.

### 3.1 Data Collection and Pre-processing

In order to conduct training, validation, and testing of the proposed approach, we have combined two publicly accessible databases, namely ACRIMA [29] and ORIGA [30]. This merging process resulted in the creation of a new dataset we've named “ORICRIMA”. It was necessary due to the data-intensive nature of DL models. Using a limited quantity of images has been recognized to adversely affect the model's performance.

There are seven hundred and five RFIs in the ACRIMA database. Three hundred and ninety-six images exhibiting signs of glaucoma and three hundred and nine images depicting normal instances. The majority of RFIs in this dataset were recorded from both the left and right eyes after dilation, with a specific emphasis on capturing images centered around the OD. A portion of these images was excluded due to the presence of artifacts, noise, and insufficient contrast. The images, captured with a 35° field of view, were offered in varying resolutions and were acquired using the IMAGEnet capture system, a digital imaging platform developed by Topcon to capture, store, and analyze high-quality RFIs with precise detail for clinical use, and the Topcon TRC retinal camera. Each image within this database received annotation by two glaucoma specialists, each possessing 8 years of expertise.

The ORIGA dataset is derived from a portion of the Singapore Malay Eye Study data, which was gathered between 2004 and 2007 through the efforts of the Singapore Eye Research Institute, with financial support from the National Medical Research Council. It consists of 482 images representing healthy conditions and 168 images depicting glaucoma, sourced from Malay adults aged 40 to 80.

The ACRIMA images were initially cropped around the OD. In contrast, the ORIGA images were initially supplied in a full fundus view. Consequently, we needed to perform cropping on the ORIGA images to make them compatible with the ACRIMA images, as illustrated in Fig. (**1**). Manual cropping aligns with the clinical aspect of our research. Glaucoma primarily affects the region around the OD and its vicinity. Moreover, by manually selecting this specific area, we ensure that our analysis focuses on the most relevant anatomical region for GD. This approach respects the clinical context and mirrors the actual diagnostic process used by ophthalmologists. Additionally, research by Orlando *et al.* [31] demonstrates the efficiency of this approach compared to using the full-fundus view images in CNN-based GD. Additionally, by focusing attention on the relevant area, this technique enhances GD, streamlining analysis, and eliminating unnecessary data, thereby reducing complexity. Automated cropping methods may not always accurately identify the OD or may inadvertently include irrelevant parts of the RFI. In contrast, manual cropping allows for precise and targeted selection, reducing the risk of including unnecessary or irrelevant information. After both datasets were merged, we had 564 images for glaucoma and 791 images for normal controls.

Next, we adopted image normalization to create a consistent data distribution, achieved by dividing the images by the channel count. Consequently, this process yields normalized data within the 0 to 1 range, ensuring a more consistent and stable training experience for DL models. Additionally, a consistent input size of 128×128×3 was applied to all models considered in this experiment. This choice of uniform image size was made strategically to optimize computational efficiency and minimize complexity.

### 3.2 Backbone

This section describes the backbone architectures utilized for feature extraction, comprising the proposed Multi-branch CNN and the enhanced adaptations of MobileNet and DenseNet201, specifically optimized for GD.

#### 3.2.1 Proposed Multi-branch CNN Architecture

The process of training a customized CNN from scratch entails constructing and training a neural network model with weights and biases that are initialized randomly. The model acquires knowledge about patterns, features, and representations only from the given dataset without dependence on preexisting knowledge or parameters. Conventional CNN architectures provide proficient performance in fundamental image classification tasks. However, they have difficulties in dealing with complex medical images that exhibit numerous diverse and detailed aspects. In order to address these constraints, the Multi-branch CNN model was developed. The current approach has been developed to improve the network's capacity for extracting features from RFIs. The architectural design of the Multi-branch model functions as one of the three base models for the BO stage. The structure of the proposed Multi-branch CNN is displayed in Fig. (**2**).

The input layer functions as the entry point for the ORICRIMA dataset. We employed RFIs with specific dimensions, precisely 128×128 pixels, and encompassing three color channels. These images initially existed in a larger image size, and the reduction of dimensions had a crucial role in reducing computing complexity.

The essential aspect of this model is the utilization of two branches, each containing its own set of convolutional and pooling layers [32, 33]. These branches function concurrently, individually processing the incoming data, thereby allowing the model to capture a wide range of features. The architectural design of the CNN involves making particular decisions about the number of filters, filter size, pool size, and stride. These choices are crucial and depend on various important parameters.

In Branch 1, the network begins with a 3×3 convolutional layer, including 32 filters. This option is appropriate for the initial layer due to its ability to capture intricate data details and its moderate capacity for feature extraction. Following that, a 2×2 max-pooling operation is employed with a stride of 2 in order to decrease spatial dimensions from 128×128 to 64×64 pixels and mitigate the risk of overfitting. Additionally, an extra 3×3 convolutional layer is introduced, consisting of 64 filters, to further strengthen the representation of features. This results in an output tensor of dimensions 32×32×64.

Branch 2 employs a comparable approach but with modifications customized to fulfil the distinct criteria of this particular branch. The initial convolutional layer has been strengthened by incorporating 64 filters, which enables the capture of more detailed features. The preservation of overall symmetry is achieved by employing 2×2 max-pooling with a stride of 2, followed by a convolutional layer containing 128 filters. This leads to the generation of an output tensor with dimensions measuring 32×32×128. The architectural decisions used in this study are intended to achieve an appropriate balance between the ability to extract features, computational efficiency, and the hierarchical representation of features inside the CNN architecture. These choices demonstrate a thoughtful approach to addressing the complexity of the dataset and the overall design principles of the network.

After the input data is processed independently by both branches, their outputs are combined by concatenating them along the channel axis. The merging of various features retrieved by each branch is a crucial step in generating a thorough representation of the input data. In order to augment the process of feature extraction, we proposed the incorporation of an extra convolutional block subsequent to the concatenation step. This block comprises a convolutional layer with a size of 3×3, consisting of 256 filters. In addition, the network incorporates max-pooling (reducing from 32×32 to 16×16 pixels) and BN techniques, which enhance its capacity to collect high-level information [34].

Our emphasis lies only on introducing the feature extraction module of the Multi-branch CNN. In our experimental setup, we've implemented the BO strategy to automatically generate an optimal classification block for this proposed network. This approach enables us to efficiently optimize the classification parameters and achieve superior performance outcomes.

#### 3.2.2 Pretrained MobileNet Architecture

MobileNet is a CNN design proposed by Howard *et al.* [35] that addresses the issue of excessive computer resources. The central architecture relies on depthwise separable convolutions, a technique that can be described as a way to simplify complexity by breaking it down into two components: depthwise complexity and a 1×1 complexity, referred to as pointwise complexity. Generally, the main distinction between the MobileNet architecture and the conventional CNN architecture lies in the utilization of a convolutional layer or a layer with a filter size that matches the dimensions of the input image. The network incorporates two straightforward global hyperparameters to effectively manage the trade-off between delay and ACC. The purpose of the width multiplier α is to uniformly reduce the network's size at each layer. Similarly, the resolution multiplier ρ is applied to decrease the dimensions of both the input image and the internal representations of each layer by the same factor. For a feature map with dimensions D_F_×D_F_, where the kernel size is D_K_×D_K_, the input channel count is *M*, and the output channel count is *N*, the overall computation, denoted as C_M_, for the core layers of the model can be expressed using Eq. (**1**).

**Table:** 

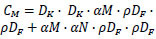	(1)

In our approach, we aim to transfer knowledge from the source domain D_s_ which consists of natural images, to our target domain of RFIs, denoted as D_T_. This entails training a target classifier, denoted as h_T_(X_T_) in such a way that when given an RFI *X_Ti_* from the target domain *D_T_*, it provides a prediction in one of two categories: *Y_Ti_={Glaucoma, Normal}*. This prediction is represented as y_*Ti*_ and is expressed in Eq. (**2**) for a specific sample *i*. Here, TL serves as a feature extractor, implying that all layers are kept fixed, and only the top layer from the source classifier *h_T_(X_T_)* is retrained to accommodate the new target classes [36].

**Table:** 

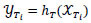	(2)

In this paper, we retained the feature extraction component of the pretrained MobileNet and made adjustments to the classification segment by incorporating our customized layers. The BO phase played an important role in determining the optimal hyperparameter values for this classification block. In order words, the MobileNet's classification block will be automatically generated to align with the requirements of this particular GD task. We tuned 6 key hyperparameters within this block, encompassing the number of dense blocks to employ, the number of dense nodes, the dropout rate, the decision of whether or not to employ BN, the flattening approach, and the choice of activation function. This approach resulted in the creation of an improved MobileNet configuration that operates efficiently for our detection task. The structure of the customized MobileNet is shown in Fig. (**3**).

#### 3.2.3 Pretrained DenseNet201 Backbone Architecture

DenseNet is a pretrained DL model that employs a feedforward approach to establish connections between each layer and all the subsequent layers. DenseNet201 takes advantage of its compact network structure, making it straightforward to train and highly parameter-efficient. This is achieved by enabling various layers to reuse features, enhancing diversity in the input of subsequent layers, and thereby enhancing overall performance [37].

We made similar modifications to DenseNet201 as we did with MobileNet, ensuring a consistent approach. Furthermore, we contemplated the optimization of identical hyperparameters within the classification block of DenseNet201. This uniformity in our methodology enabled us to refine both models harmoniously, addressing specific requirements for our task effectively. The structure of the customized DenseNet201 is displayed in Fig. (**4**).

### 3.3 BO for Hyperparameter Tuning

In this paper, we employ BO to automate the selection of the most suitable hyperparameter values for our three models: Multi-branch, MobileNet, and DenseNet201. The global optimization problem involves the quest to discover an input that minimizes or maximizes a given objective cost function across all feasible inputs. Frequently, this objective function proves difficult to assess due to its non-linear, non-convex, noisy, high-dimensional, and computationally demanding nature. BO is adept at addressing global optimization challenges, with its foundation lying in Bayes's theorem [38]. Furthermore, using prior knowledge, Bayes allows for the estimation of the posterior distribution. Eq. (**3**) depicts the general form of the Bayes theorem.

**Table:** 

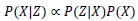	(3)

The posterior distribution (*P*(*X*|*Z*)) is directly related to both the likelihood (*P*(*Z*|*X*)) and the prior distribution (bias) (*P*(*X*)). BO updates posterior knowledge based on new information about the objective function, which improves ACC and decreases model loss. As part of the optimization process, it uses a surrogate network that is then fitted to the recorded findings of the actual network. In this context, a finding represents a fully trained version of one of our CNNs with specified hyperparameters. For each iteration, a set of hyperparameters is chosen, and then an observation is made. The observation's evaluation is determined relying on the validation ACC. The hyperparameter set is selected with the help of an acquisition function that tries to find a good balance between surveying the entire search dimension and focusing on regions that work well. In our study, we employed the Expected Improvement [39] acquisition function. This function assesses the anticipated enhancement a point can offer when investigating the neighborhood of the current best value. If the function's improvement falls short of the expected value following the algorithm's execution, it suggests that the current optimal point might be a local solution, prompting the algorithm to explore other areas in the domain to locate the optimal point. Fig. (**5**) presents an overview of the workflow of the BO strategy, while the corresponding pseudo-code is outlined in Algorithm 1.

Multiple iterations of this optimization process are conducted to tune the validation set, yielding the optimal hyperparameters for this binary classification task. In this paper, each model experiences 40 iterations. We made selections for 6 crucial hyperparameters within the classification components of both MobileNet and DenseNet201 models. These encompassed decisions regarding the number of dense blocks to utilize, the number of dense nodes, the dropout rate, whether to employ BN, the flattening type, and the selection of the activation function. In addition to these 6 parameters, we introduced a 7th parameter, which pertains to the choice of optimizer. It is important to emphasize the significance of tuning the optimizer choice as it can profoundly impact the model's convergence and overall performance. The final dense layer employing the sigmoid function for class prediction remains fixed and cannot be tuned.

**Table:** 

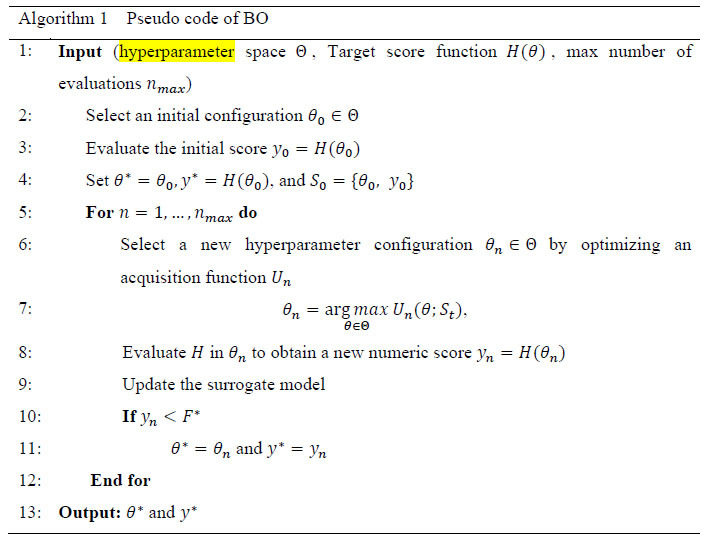

For the Multi-branch model, we followed a similar approach for tuning both the classification components and the optimizer, aligning with the strategy employed for the pretrained models. The only difference lies in our consideration of optimizing the activation function for the convolution layers, given that the feature extraction segment of the model is trainable from scratch.

### 3.4 Selection of Hyperparameters

This section details the critical hyperparameters examined to optimize the performance of the three CNN models, including the number of dense blocks, the number of nodes, dropout rate, BN, flattening type, activation function, and optimizer. The objective is to fine-tune each hyperparameter within defined ranges to achieve an optimal balance between model complexity, overfitting prevention, and efficient training.

#### 3.4.1 Number of Dense Blocks

Each dense block in the classifier must consist of a dense layer [40], a dropout layer, and the option to include a BN layer or not. Furthermore, by exploring a range of 1 to 5 dense blocks, we aim to assess how the complexity of the classification architecture influences the model's classification performance. Insufficient complexity in the classification part, represented by fewer dense blocks, can lead to underfitting, where the network is unable to discern crucial insights in the images. On the flip side, an abundant number of dense blocks can lead to overfitting.

#### 3.4.2 Number of Dense Nodes

Determining the overall architecture of our CNN relies heavily on the number of nodes selected for the dense layers. These layers do not come into direct contact with the outer environment, however, they still have a major influence on the result. When fewer nodes are employed in the dense layers, an issue known as underfitting occurs, and vice versa. The aim of this paper is to find the optimum number of nodes needed for each of the three CNNs to perform to their best capabilities. For this hyperparameter, we fix the range between 32 and 1024.

#### 3.4.3 Dropout Rate

Dropout [41] is a regularization method used to mitigate the overfitting issue. The process involves removing a group of neurons from the structure temporarily. The dropout-rate search dimension is set between 0.2 and 0.5. Within this range, we explore both moderate (0.2) and more aggressive (0.5) dropout strategies, acknowledging that the optimal dropout rate can differ based on the model's complexity and the dataset.

#### 3.4.4 BN

In deep neural networks, there is a continual shift in the distribution of inputs to the dense layers during training. This phenomenon is referred to as “internal covariate shift”. Typically, this evolving distribution gradually approaches the upper and lower bounds of the activation function's range. Consequently, this can result in a decrease and eventual vanishing of gradients in shallow, dense layers during the backpropagation process. This is a key reason for the progressively slower convergence observed in deep neural networks. BN has the ability to transform the input distribution into a standard normal distribution characterized by a mean of 0 and a variance of 1 [42, 43]. During this optimization phase, we aim to ascertain the advantages of employing both BN and dropout layers within each dense block of our three CNN models. We seek to determine whether the combination of these techniques brings notable benefits to the performance of our models.

#### 3.4.5 Flattening Type

The cornerstone of constructing effective CNN models lies in comprehending how different layers and functions influence the overall performance of our model. In pursuit of this understanding, our experiment delved into the evaluation of both flattening and GAP to ascertain which method aligns optimally with each of our three models [44]. The purpose of this exploration was to determine of these various approaches which one best fits the special needs and performance targets of our three models.

#### 3.4.6 Activation Function

The activation function is essential for building a CNN. It uses the weighted sum and an additional bias to determine whether a neuron should be active. In order to learn and carry out more complex tasks, the activation function performs a non-linear transformation on the input. The range explored for the activation functions during this optimization encompasses ReLU, ELU, Tanh, Swish, and Softplus functions [45-49].

#### 3.4.7 Optimizer

An optimizer is a method for finding the optimal value of an objective function in an optimization problem. Gradient descent strategies are the most significant and extensively utilized methods for enhancing models used in DL [50-53]. Because of the massive size of the database and the limited processing memory, especially on GPUs, the traditional batch gradient descent methodology and the stochastic gradient descent method are not suitable for performing training efficiently and effectively. In order to find a solution, this BO experiment tried out several different gradient methods. Nadam [54], RMSProp [55], Adam [56], SGD [57], and AdaMax [58] are the optimizers that have been specified. Table **1** outlines the hyperparameters for the BO experiment along with their corresponding search ranges.

### 3.5 Proposed Dirichlet-based WAE

Improved generalization performance can be achieved through EL, a method that combines multiple individual models. It's widely recognized that DL models outperform traditional ones. To further enhance generalization, deep EL models combine the strengths of both DL and EL. By merging numerous deep models (base-learners) into a single model, we can create a superior model that benefits from the knowledge in the majority of the training data. Different EL strategies, such as max voting, averaging, weighted averaging, and more, can be employed to improve model performance. Our paper is dedicated to the challenging task of improving the ACC of GD in RFIs. To achieve this, we've adopted an advanced method called the WAE [59]. This approach combines the predictive abilities of three different CNN models: MobileNet, DenseNet201, and a Multi-branch CNN model. What's unique about our work is that we've combined the concepts of TL and training custom CNN from scratch, marking a significant contribution to the GD methodologies. The WAE strategy represents a refined approach within EL. It leverages the distinct strengths of each individual model while effectively addressing their limitations.

The selection of models within the ensemble plays an important role in our approach. Each model contributes a distinct viewpoint, similar to assembling a team of experts with a wide range of skills. MobileNet is renowned for its efficiency in terms of model size and computational demands. Its specialization lies in capturing intricate details within images, a valuable attribute for detecting subtle indications of glaucoma. On the contrary, DenseNet201 represents a different CNN architecture. It excels in capturing intricate, hierarchical features in images. This model focuses on comprehending the broader context within RFIs, a crucial aspect for precise diagnosis. The Multi-branch CNN model adds another layer of diversity. It is designed to merge two branches of convolutional layers, effectively offering multiple perspectives on the same image. This diversity empowers the ensemble to encompass a wide spectrum of image features and patterns that directly pertain to GD. By incorporating these diverse models, our ensemble approach harnesses the collective potency of their distinct capabilities, equipping it to handle the complexities and variations inherent in RFIs effectively.

Let's delve deeper into the concept of WAE. Picture each model as an expert, each with its unique specialization. MobileNet, DenseNet201, and Multi-branch each bring their distinctive insights to the task of GD. Now, the WAE functions like a council of experts, taking into account the perspectives of these models but assigning varying degrees of importance to each expert's input. The mathematical formulation of WAE ensures that no single model dominates the decision-making process. Instead, it follows a cooperative approach where the ensemble's collective prediction, *Ŷ_E_* relies on assigning suitable weights (*Ŷ*_1_, *Ŷ*_2_, and *Ŷ*_3_) to each model's individual predictions (*w*_1_, *w*_2_, and *w*_3_). The mathematical expression of this concept is expressed by Eq. (**4**).

**Table:** 

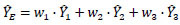	(4)

Here, the values of *w*_1_, *w*_2_, and *w*_3_ establish the level of influence that each model has on making the final prediction. This weighted combination empowers the ensemble to harness the distinct capabilities of each model, thereby yielding a GD system that is more resilient and precise.

Now, let us shift our focus to a novel and mathematically rigorous facet of our approach: the exploration of weights through the utilization of the Dirichlet distribution [60]. This is the key to determining the significance that each model should hold within the ensemble. The Dirichlet distribution acts as our guiding mathematical instrument. It is defined by a probability density function, which may seem intricate initially but serves a crucial role in guaranteeing the validity and equilibrium of our weight assignments. The function is formally represented as (Eq **5**):

**Table:** 

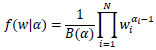	(5)

Here, the vector *w* signifies the set of weights we intend to sample. In our context, this vector comprises three components: w_1_, w_2_, and w_3_. *N* represents the number of models or experts in the ensemble. The parameters α_i_ govern the configuration of the distribution, determining the probability of sampling particular weight combinations. The power of this method is its systematic approach to investigating weight combinations. It guarantees the validity of weights, ensuring they remain within the range of 0 to 1 and sum up to 1. This careful exploration process makes it possible to pinpoint the best weight distribution to get the maximum effect from the ensemble. Our method involves an iterative sampling process with all possible weight combinations drawn from the Dirichlet distribution over 200 trials. In each trial, we compute the ensemble prediction *Ŷ_E_* by employing the weights drawn from the Dirichlet distribution. Validation ACC is the main metric that we use to evaluate the performance of each weight combination. This metric provides insight into the performance of our ensemble model on a set of images set aside for validation. The flow of our proposed Dirichlet-based WAE is shown in Fig. (**6**).

### 3.6 Fine-tuning of Improved MobileNet and DenseNet201 Architectures

After optimizing the classification blocks of the pretrained MobileNet and DenseNet201 architectures using BO, we proceeded to further improve model performance through a fine-tuning approach. This approach enables the model to transfer previously learned features, while adjusting the later layers to capture task-specific patterns. This section presents the mathematical formulation of the fine-tuning strategy and its implementation for MobileNet and DenseNet201.

#### 3.6.1 Mathematical Framework of Fine-tuning

Let *f(x;θ)* denote the pretrained model, where *x* represents the input image and *θ* corresponds to the model parameters, which include both weights and biases. The model is structured into multiple layers, 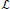
_1_, 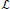
_2_,…,
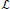
_*N*_, with associated parameters *θ_i_* for each layer, where *i* ϵ {1, 2, …, *N*}.

• **Frozen layers:** In the fine-tuning process, certain layers are “frozen”, meaning their parameters are fixed and do not experience updates during training. For these layers, the gradient update is zero, *i.e*., ∆*θ_i_* = 0 for all *i* ϵ 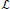
_ƒ_ , where 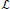
*_ƒ_* denotes the set of frozen layers.

• **Unfrozen layers:** Other layers are “unfrozen”, and their parameters are updated during training based on the task-specific data. The update rule for the parameters of an unfrozen layer 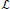
_u_
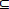

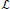
is given by:

**Table:** 

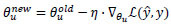	(6)

Where θ_u_^*old*^ refers to the initial parameters of the unfrozen layer, θ_u_^new^ represents the updated parameters, *η* is the learning rate, determining the step size of the update, 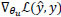
is the gradient of the loss function 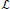
with respect to the parameters of the unfrozen layer, computed via backpropagation, *ŷ* is the model's predicted output, and *y* is the true label. In the context of binary classification for GD, the loss function 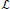
is the binary cross-entropy loss:

**Table:** 

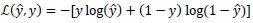	(7)

This loss quantifies the discrepancy between the predicted and actual labels, guiding the parameter updates during training.

#### 3.6.2 Fine-tuning for MobileNet

For the MobileNet architecture, fine-tuning was implemented by selectively freezing and unfreezing specific layers. In particular:

• **Frozen layers:** The first 5 depth-separable convolution blocks were frozen, as these layers capture low-level features, such as edges, textures, and basic shapes, which are generally applicable across various visual recognition tasks. Freezing these layers helps prevent overfitting and reduces computational overhead.

• **Unfrozen layers:** Layers from the 6th to the 13th depth-separable convolution blocks were unfrozen to allow the model to adapt to higher-level, task-specific features related to GD. These layers are responsible for more complex feature extraction that is directly relevant to the target task.

The updates to these unfrozen layers are calculated using the gradient descent rule as described in Eq. (**6**).

#### 3.6.3 Fine-tuning for DenseNet201

A similar fine-tuning approach was applied to the DenseNet201 architecture, which is known for its densely connected layers that promote feature reuse. The fine-tuning strategy for DenseNet201 was as follows:

• **Frozen layers:** The first convolutional layer through transition layer 2 was frozen, as these layers extract general features that do not require adaptation for the specific task of GD. Freezing these layers preserves the core feature extraction capabilities.

• **Unfrozen layers:** Starting from Dense Block 3 and continuing to the final layers of the feature extraction part, the layers were unfrozen. These layers are responsible for extracting high-level features that are more specific to our detection task.

The fine-tuned MobileNet and DenseNet201 architectures are displayed in Fig. (**7**). The fine-tuning strategy is informed by the outcomes of BO, guaranteeing that the modifications implemented during fine-tuning are consistent with the most effective hyperparameter settings. This provides a structured and systematic methodology for model refinement.

### 3.7 Performance Evaluation Metrics

In healthcare applications, image classification holds a significant position, particularly for the detection and prediction of the severe condition of glaucoma in RFIs. There exist multiple approaches for assessing classifier performance; however, we have chosen to utilize metrics derived from the confusion matrix for validation purposes [61]. These performance metrics encompass ACC, PREC, SEN, and F1, all of which serve as measures to evaluate the predictive efficacy of our proposed models [62-64]. The metric considers both false positives and false negatives, making it particularly advantageous in the context of imbalanced datasets. Eqs (**8**-**11**) display the mathematical representations of these metrics.

**Table:** 

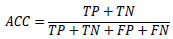	(8)

**Table:** 

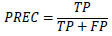	(9)

**Table:** 

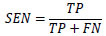	(10)

**Table:** 

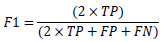	(11)

In this context, TP stands for True Positives, TN represents True Negatives, FP describes False Positives, and FN denotes False Negatives.

### 3.8 Experimental Setup

All experiments were carried out on Google Colab [65], a cloud-based platform that provided access to a T4 GPU, offering substantial computational resources necessary for both DL model training and optimization. The available 12GB of RAM further supported efficient dataset processing and computation. For hyperparameter optimization, we employed BO via the Scikit-optimize library in Python, selected for its effectiveness in exploring high-dimensional search spaces and optimizing complex models. The development and training of DL models were conducted using TensorFlow [66] and Keras [67] frameworks, selected for their adaptability and comprehensive tools supporting model construction and training. TensorFlow and Keras also efficiently utilized the GPU resources on Google Colab, facilitating accelerated training and iterative experimentation.

## 4 RESULTS

In this section, we show our proposed framework to be effective in GD from RFIs. Our ORICRIMA dataset, comprising 1,355 RFIs, was divided into three subsets: training (70%), validation (15%), and testing (15%). With a training set of 948 samples, data was input into the model to extract valuable insights. A validation set of 203 samples was set aside for tuning hyperparameters and observing performance. The testing set of 204 samples continued to stand alone; it provided the ultimate overall assessment of our deep models.

### 4.1 Convergence Analysis

Fig. (**8**) shows the convergence curves for MobileNet, DenseNet201, and the Multi-branch models, illustrating how validation ACC improved over 40 iterations in the BO process. Each iteration had a training duration of 16 epochs to demonstrate that our optimization strategy can achieve accurate gradient descent with minimal training. The initial hyperparameters were randomly selected to highlight the efficacy of the optimization process. MobileNet's curve shows notable early gains in validation ACC, indicating that the BO process effectively identifies optimal hyperparameters, leading to significant detection improvements. However, as iterations continue, the validation ACC stabilizes, indicating diminishing returns from further optimization. Similarly, DenseNet201's convergence plot shows significant early improvements in validation ACC, but these gains level off with continued optimization. This pattern suggests that the chosen hyperparameters achieve a balance between model complexity and GD ACC. The Multi-branch CNN demonstrates a similar pattern: early rapid improvements in validation ACC followed by a plateau, suggesting that the hyperparameters effectively fine-tune the model without further need for optimization. The choice to conduct 40 iterations was justified, as fewer iterations might have missed these initial improvements, particularly for MobileNet.

### 4.2 Impact of the Proposed Optimization Strategy on the Backbone Models

Table **2** compares our DL models' performance before and after hyperparameter tuning. Initially, MobileNet, DenseNet201, and the Multi-branch model showed varying ACC, with 81.77%, 81.28%, and 43.84%, respectively. After hyperparameter tuning, RMSProp improved MobileNet, Adamax worked well for DenseNet201, and SGD enhanced the Multi-branch model. The use of BN also played a role, with it benefiting MobileNet but not the other two models. Other architectural changes, like the number of dense blocks and nodes, boosted DenseNet201's ACC to 92.61% and the Multi-branch model's to 94.08%. The Multi-branch model's ACC improvement by over 50% after tuning underscores the potential of customized DL configurations.

Fig. (**9**) shows the distribution of dense blocks among the three CNN models. A dense block consists of a dense layer, a dropout layer, and, optionally BN. MobileNet typically uses one to four dense blocks, with one block being the most common configuration. This setup achieved 89.16% ACC, indicating a preference for simplicity in MobileNet's architecture. DenseNet201 has a more varied approach, with one to five dense blocks. The most frequently chosen setup has five dense blocks, achieving 92.61% ACC, suggesting that more complex architectures can be beneficial for DenseNet201. The Multi-branch model shows a mix of simplicity and complexity, with the optimal setup having three dense blocks. This configuration resulted in 94.08% ACC, indicating a balance between simple and complex structures.

Fig. (**10**) illustrates the distribution of dense node counts for each model. MobileNet typically uses 32 dense nodes, matching its preference for straightforward architectures. DenseNet201 commonly uses 1024 dense nodes, but the optimal setup has 797 nodes, suggesting that a moderate level of complexity works best. The Multi-branch model tends to favor around 1024 dense nodes, but the optimal configuration is 821 nodes, indicating the benefit of a moderately complex design.

In Fig. (**11**), the distribution of dropout rates is shown. MobileNet's most common dropout rate is 0.5, consistent with its optimal configuration. DenseNet201 also commonly uses a 0.5 dropout rate, but the optimal rate is 0.207, indicating that a lower dropout rate can be more effective. The Multi-branch model frequently uses a dropout rate of 0.2, which is also its optimal configuration, suggesting a more cautious approach to avoid overfitting.

Fig. (**12**) presents the use of BN in the three models. MobileNet has a balanced distribution, indicating flexibility in applying BN. The optimal setup involves enabling BN, which improves performance. In DenseNet201, BN is often turned off in dense blocks, which aligns with its design principle of feature concatenation. The Multi-branch model also typically disables BN in its dense blocks, consistent with DenseNet201.

Fig. (**13**) shows the preferences for flattening layers. The optimal setup in all three models involves flattening layers, which convert feature maps into one-dimensional vectors, aiding in classification. GAP is occasionally considered, suggesting some adaptability in the optimization strategy.

Fig. (**14**) depicts the preferences for activation functions. ELU is the most commonly chosen across all three models, but BO recommends ReLU for DenseNet201 and the Multi-branch model, indicating a preference for simpler and more efficient activation functions. MobileNet benefits from ELU's flexibility.

Fig. (**15**) shows the distribution of optimizer choices. MobileNet prefers Adam and Nadam, but the optimal choice is RMSprop, which adapts to its lightweight structure. DenseNet201 often uses Adam, but BO recommends Adamax, which handles sparse gradients effectively. The Multi-branch model leans toward SGD, and BO confirms this as the optimal choice, indicating that its complex architecture benefits from SGD's robustness. The analysis demonstrates that different hyperparameter choices align with each model's architecture. BO helps find the optimal configurations, leading to significant improvements in model ACC. This customization across different hyperparameters emphasizes the importance of customizing the optimization process to each model's unique requirements.

### 4.3 Partial Dependence Analysis

**Fig. (16):**
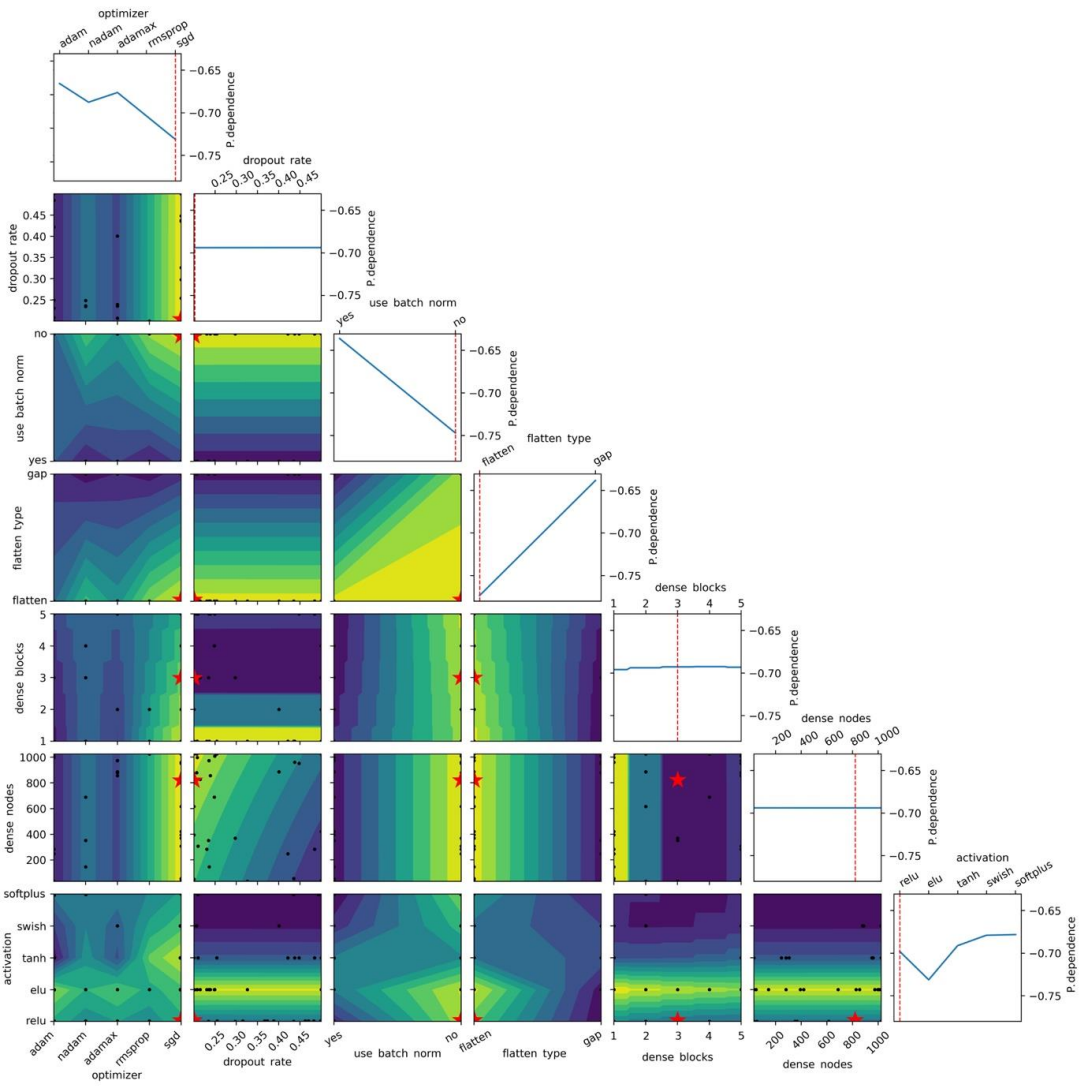
Partial dependence plots of Multi-branch CNN. P.dependence: Partial dependence.

Moreover, to understand the impact of BO on model performance through hyperparameter tuning, we used partial dependence plots to analyze the Multi-branch model, which showed the greatest improvement in ACC after optimization (Fig. **16**). These plots focus on individual hyperparameters while keeping the others at their optimal values, allowing us to see how each hyperparameter affects the objective function. The dashed red lines indicate the optimal settings, while black data points represent the sampled hyperparameter combinations. Red stars highlight the best choices, yellow outlines mark hyperparameters linked to better performance, and blue outlines indicate those associated with reduced performance. It is essential to recognize that the negative values displayed on the y-axis do not inherently convey a negative meaning. Instead, they represent the response of the objective function to various hyperparameter settings. In terms of optimizer choice, SGD yielded the most favorable outcomes, with values around -0.74. Dropout rates hovered around -0.69, with an optimal rate of 0.2. BN had mixed results: using BN “yes” resulted in values around -0.63, while not using BN “no” had values near -0.75, with the boundary favoring the “no” setting. The choice between flattening layers and GAP influenced the objective function, with flattening yielding values around -0.77 and GAP around -0.63, indicating a preference for flattening layers. The optimal number of dense blocks was typically three, with values centered around -0.69. The ideal number of dense nodes was 821, with similar values. When examining activation functions, ReLU had slightly higher values, around -0.70, while ELU and Tanh were lower at -0.73 and -0.69, respectively. Swish and Softplus were comparable, each at around -0.67, with the boundary favoring ReLU. These findings from the partial dependence plots align with our earlier results, confirming the effectiveness of BO in guiding hyperparameter tuning for improved model performance.

### 4.4 Impact of the Proposed Dirichlet-based WAE on the Performance

**Fig. (17):**
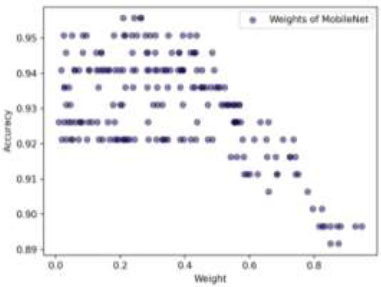
Weight distribution for MobileNet model.

**Fig. (18):**
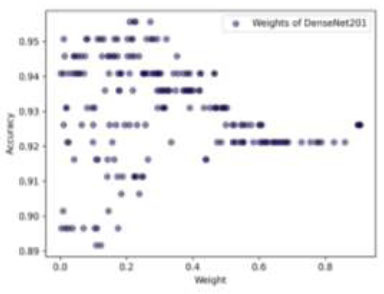
Weight distribution for DenseNet201 model.

**Fig. (19):**
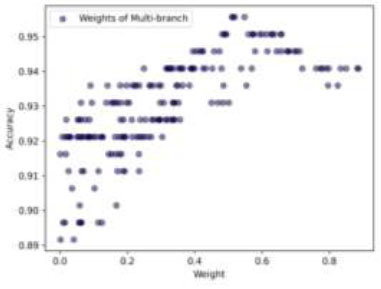
Weight distribution for the multi-branch model.

Additionally, to improve the performance of our three optimized CNN architectures, we used a Dirichlet-based WAE method. This proposed technique explores 200 different weight combinations to determine the best weights for each model. Moreover, by using the Dirichlet distribution, we can fine-tune each model's contribution, aiming to enhance the overall performance of the ensemble. We carefully sample weights with four decimal places of PREC, allowing us to adjust the models' contributions very accurately. For example, a weight could be 0.1234, where the last four digits reflect the decimal PREC. This level of detail is crucial for finding the best combination of weights. (Fig. **17**) illustrates the weight distribution for MobileNet. If we reduce its weight, the ensemble's ACC improves; if we increase it, the ACC drops. This happens because MobileNet's individual ACC is relatively low at 89.16%. The optimal weights for MobileNet are approximately (0.2092, 0.2431, 0.2624, 0.2647), showing the importance of fine-tuning MobileNet's contribution. DenseNet201 shows a different pattern (Fig. **18**). It tends to perform better with lower weights, suggesting that too much emphasis on DenseNet201 can negatively impact the ensemble's ACC. Ideal weights for DenseNet201 are around (0.2093, 0.2219, 0.2270, 0.2719), indicating that a moderate contribution is optimal. The Multi-branch model (Fig. **19**) has a direct relationship between weight and ACC. Higher weights lead to better performance, in line with its individual ACC of 94.08%. However, overemphasis on the Multi-branch model can slightly reduce ensemble performance. The best weights for the Multi-branch model are about (0.5104, 0.5132, 0.5187, 0.5475), suggesting the need for a balanced approach.

The four optimal weights for our models come from iterations 79, 120, 134, and 169, leading to an ensemble ACC of 95.56%. Table **3** outlines key performance metrics, highlighting the ensemble's superior PREC, a crucial measure for GD. For example, while MobileNet achieves a PREC of 84.78%, the ensemble boosts it to 96.39%, reducing FP. The ensemble also excels in SEN, which is essential for detecting true glaucoma cases. DenseNet201 has an SEN of 94.87%, but the ensemble increases it to 97.44%, indicating its reliability in accurately identifying glaucoma cases without overlooking normal ones. This balance is vital in medical diagnosis, avoiding both FP and FN. The F1 for normal cases is another success for the ensemble, with a score of 95.44% compared to the Multi-branch model's 95%. This demonstrates the ensemble's ability to maintain a balance between PREC and SEN. The ensemble's AUC of 0.9877 demonstrates its excellent discriminatory power, crucial for accurate diagnosis. This high score reflects the ensemble's capacity to distinguish between normal and glaucoma cases, a key factor in clinical outcomes.

### 4.5 Evaluation on the Unseen Images

After validating our DL models, we moved to the testing phase to evaluate performance in real-world scenarios. Using a separate set ensured independent results, free from any data contamination or bias. This independent test set helped us understand how well our ensemble model adapts to new, unseen data. Table **4** provides a summary of the results, which, while slightly lower than in the validation phase, still underscore the value of our approach. The ensemble model, combining multiple DL architectures, achieved impressive performance during testing. It reached a PREC of 91.44%, a SEN of 91.56%, an F1 of 91.49%, an ACC of 91.66%, and an AUC of 0.9692. These results confirm that the ensemble model is effective for GD. Notably, while the Multi-branch model outperformed DenseNet201 and MobileNet during validation, DenseNet201 slightly surpassed the Multi-branch model in the test phase, with an ACC of 90.19% compared to 88.72%. This emphasizes the importance of testing with an independent dataset to reflect real-world conditions.

The ROC curves are the basis for assessing our DL model's ability to separate between glaucoma and non-glaucoma cases. These curves show that the curves demonstrate the ACC of the model by plotting TP and FP rates. In Fig. (**20**), the ROC curves of MobileNet, DenseNet201, the Multi-branch model, and the ensemble model are compared. MobileNet ROC curve, with an AUC of 0.9141, shows that it can differentiate between glaucoma and non-glaucoma cases. Nevertheless, its average slope might possibly result in some extra FP because of its simpler structure. DenseNet201's ROC curve is steeper, indicated by an AUC of 0.962, suggesting that this network has a stronger capacity to differentiate between positive and negative cases, thereby generating less FP. Multi-branch model's ROC curve is not far from DenseNet201, but has a slightly lower AUC of 0.9605. This shows that the Multi-branch model can differentiate glaucoma cases almost as well as DenseNet201, thus adding value to the entire ensemble. The ensemble model is the model with the highest AUC value (0.9692), which makes it the best-performing model. Its steep ROC curve indicates it can distinguish glaucoma and non-glaucoma cases well, and this shows the strength of ensemble modeling.

### 4.6 Effect of Fine-tuning

In addition, to further enhance the performance of the proposed ensemble, fine-tuning was applied to the optimized MobileNet and DenseNet201 models. This fine-tuning strategy was not employed for the Multi-branch model, as it was trained from scratch without leveraging TL, in contrast to the MobileNet and DenseNet201 models. In addition, the number of training epochs was increased from 16 to 32 for all three models to promote improved convergence and enhance model generalization. The fine-tuning procedure utilized the hyperparameter configurations identified during the BO phase. By fine-tuning, the pretrained weights of MobileNet and DenseNet201 were adjusted to better capture task-specific features while preserving the general features acquired from large-scale datasets (*e.g*., ImageNet) during their initial training. Following fine-tuning, the Dirichlet-based WAE ensemble method was employed again to combine the three models. Table **5** presents the impact of fine-tuning and the extended training epochs on the performance of the individual models and the Dirichlet-based WAE ensemble model, evaluated on both the validation and test sets. The results indicate that both fine-tuning and extending the training duration positively influenced model performance. Fine-tuning with 32 epochs led to substantial improvements across all models. In particular, for MobileNet and DenseNet201 models, fine-tuning not only enhanced overall performance on the validation and test sets but also ensured the retention of generalization capabilities while adapting the models more effectively to the specific requirements of GD. Although the Multi-branch model was not fine-tuned, slight performance improvements were observed with the extended training duration. The Dirichlet-based WAE ensemble model, which integrated the fine-tuned MobileNet, DenseNet201, and Multi-branch models, achieved the highest performance, demonstrating that the ensemble approach effectively enhanced both ACC and robustness.

### 4.7 Comparison with Existing Approaches

Furthermore, to thoroughly evaluate our framework, we compare the performance of the four proposed models (Improved MobileNet, Improved DenseNet201, Improved Multi-branch CNN, and their ensemble) against recent works on GD from RFIs. Different studies have utilized a range of datasets, as presented in Table **6**, with some authors combining multiple datasets while others have evaluated their models using individual datasets. These methods can be categorized into four distinct groups. The first group consists of approaches that manually extract features using techniques such as QB-VMD, DWT, EWT, and VMD, followed by classification with SVM. While some of these methods achieved moderate ACC, the study by Maheshwari *et al.* [19] reported an impressive 95.1% ACC, which is competitive with the performance of our ensemble model. However, these methods separate the feature extraction and classification stages, meaning that the feature extraction step does not benefit from the model's ability to learn task-specific features. This separation limits the model’s ability to refine feature representations based on the task at hand. The second group includes methods that customize CNN architectures and rely on learning features directly from the dataset, similar to our Multi-branch CNN. Our customized CNN outperformed the models by Bajwa *et al.* [20] and Liao *et al.* [23], achieving an AUC of 0.961. However, there is still room for improvement by integrating additional branches with innovative blocks to further enhance feature extraction. The third group consists of models that utilize TL, as we did with MobileNet and DenseNet. Our improved MobileNet outperformed the MobileNetV2 model by Martins *et al.* [26] despite their use of a larger dataset. Our two improved pre-trained models achieved an ACC of up to 94%, surpassing most models in the existing literature. The fourth group includes approaches that combine multiple CNNs, similar to our Dirichlet-based WAE model. Cho *et al.* [27] generated an ensemble of 56 CNNs, while Serte *et al.* [28] proposed two ensemble combinations: a three-model combination of AlexNet, ResNet50, and ResNet152, and a dual-model combination of AlexNet and ResNet50. Our ensemble model outperformed these approaches by a significant margin, making it a compelling choice for clinical applications.

## 5 DISCUSSION

The experimentation yielded valuable insights, demonstrating that the utilization of BO for hyperparameter adjustment had a substantial positive impact on the DL models’ efficacy. The convergence curves illustrated the success of this optimization process in identifying the most advantageous hyperparameter configurations, resulting in significant enhancements in the ACC of GD. These findings underscore the importance of making meticulous selections of hyperparameters when dealing with DL models in the field of medical image analysis. Importantly, the selection of the optimizer emerged as a crucial factor influencing model performance. Distinct optimizers were identified as being more suited to particular models. For instance, RMSProp, Adamax, and SGD exhibited remarkable effectiveness when paired with MobileNet, DenseNet201, and the Multi-branch model, respectively. Here is an illustration of the idea that a one-size-fits-all strategy is perhaps not the best, pointing to the benefits of customizing hyperparameters to the unique characteristics of each individual model.

The application of the Dirichlet-based WAE method effectively allowed us to combine the strengths of multiple DL models. The precise assignment of weights, achieved through a comprehensive exploration of weight combinations, proved to be a crucial step in attaining a well-balanced ensemble. This weight distribution analysis showed that each model had its own ideal weight distribution. Additionally, the ensemble model was constantly superior to individual models across a number of performance metrics, including PREC, SEN, F1, ACC, and AUC. This highlights the substantial contribution of EL in improving the ACC of GD. Importantly, the ensemble approach excelled in reducing FP, a critical aspect in medical diagnosis, as it minimizes the risk of erroneously classifying healthy retinas as affected by glaucoma.

The fine-tuning strategy employed for MobileNet and DenseNet201 played a crucial role in further improving the performance of the ensemble model. By unfreezing certain blocks in the feature extraction layer and extending the training duration from 16 to 32 epochs, the models were able to refine their representations of glaucoma-specific features, leading to improvements in both validation and test ACCs. The fine-tuning process also ensured that the models retained generalization capabilities while adapting more effectively to the specific task of GD. Interestingly, while the Multi-branch model was not fine-tuned due to its training from scratch, slight performance improvements were still observed with the extended training duration.

A notable strength in our research is that our models are evaluated on a separate testing dataset. Therefore, the positive results we observe during validation are truly applicable to practical scenarios. Compared to the validation set, the performance metrics dropped only slightly, an expected result given the inherent variety in real-world clinical data. Importantly, our models exhibited adaptability and resilience in addressing this variability, further underscoring their potential importance in clinical applications.

Building on the encouraging outcomes of this study, several directions for future research can be pursued to advance automated GD and enhance the robustness and clinical applicability of the proposed models. One notable limitation of the current study is the reliance on a limited number of datasets (ACRIMA [29] and ORIGA [30]). Although these datasets provided valuable insights, the models' generalization capability can be improved by evaluating them on a more diverse set of data. Specifically, expanding the dataset to include RFIs from different populations and clinical settings is essential. Variability in imaging devices, acquisition conditions, and demographic factors (such as age, ethnicity, and comorbidities) can influence model performance in real-world clinical environments. Consequently, further evaluation using datasets like RIM-ONE [18], Harvard [27], and others from varied geographical regions is recommended to assess model robustness across diverse data sources. Additionally, incorporating images from different stages of glaucoma alongside normal RFIs would provide a more comprehensive assessment of the model's performance and limitations.

In addition, while this study primarily focuses on RFIs, the inclusion of other data types, such as optical coherence tomography (OCT) images, visual field tests, and patient demographic and medical history, could further enhance the model’s diagnostic ACC. A multi-modal approach, where different data inputs (*e.g*., RFIs and OCT scans) are processed in parallel branches before being fused for joint prediction, holds promise. Integrating clinical parameters, such as IOP, could also contribute valuable information to the model, improving its diagnostic capabilities.

Moreover, the performance of DL models typically benefits from larger datasets. Since acquiring additional labeled RFIs can be resource-intensive, methods such as data augmentation and synthetic data generation will be critical in future work. Exploring the use of generative adversarial networks (GANs), specifically deep convolutional GANs, for generating synthetic RFIs could help address dataset limitations. This approach would allow for the creation of realistic images that augment the training set. In addition to synthetic image generation, traditional augmentation techniques, such as rotation, flipping, and scaling, could be employed to further increase dataset diversity. Data augmentation will improve the models' robustness to variations in image orientation, scale, and lighting conditions, enhancing their performance in real-world applications.

Lastly, although BO has demonstrated effectiveness in automating hyperparameter tuning, there are additional optimization algorithms that could enhance model performance. For instance, hyperband and population-based training (PBT) represent promising optimization techniques that warrant exploration. Hyperband is a bandit-based method that dynamically allocates resources across different configurations, while PBT combines hyperparameter tuning with the training process, adjusting parameters during training. These methods could accelerate convergence and yield superior performance, especially in complex models such as deep neural networks. Moreover, employing more advanced Bayesian priors and acquisition functions in the optimization process could further refine hyperparameter selection and improve model performance.

## CONCLUSION

Glaucoma represents a serious challenge within the field of ophthalmology, often culminating in visual impairment. It manifests with eye-related symptoms and can lead to blindness if not detected early. In this study, we have thoroughly examined the use of DL models to identify glaucoma from RFIs. Our paper has concentrated on optimizing and refining CNN architectures, specifically MobileNet, DenseNet201, and Multi-branch CNN, using BO and fine-tuning techniques. We have also introduced a novel ensemble method based on WAE and Dirichlet distribution to effectively combine the predictions of these models.

The experimental findings highlight significant progress in the automated detection of glaucoma. The fine-tuned MobileNet and DenseNet201 architectures achieved classification ACC scores reaching 94%, surpassing the performance of TL-based models reported in previous studies. The integration of a Dirichlet-based ensemble method further improved diagnostic outcomes, achieving an ACC of 95.09%, PREC of 95.51%, SEN of 94.55%, an F1 of 94.94%, and an AUC of 0.9854. This approach also demonstrated enhanced reliability, particularly in minimizing FP, a crucial factor in the context of clinical diagnostics.

These results emphasize the substantial potential of DL technologies in advancing GD. Through the integration of advanced optimization frameworks and innovative ensemble techniques, this study establishes a new benchmark for developing cutting-edge diagnostic systems for ophthalmic diseases. Future research could build upon these findings by incorporating multi-modal data, enhancing model interpretability, and addressing broader challenges to further improve automated systems for GD.

## Figures and Tables

**Fig. (1):**
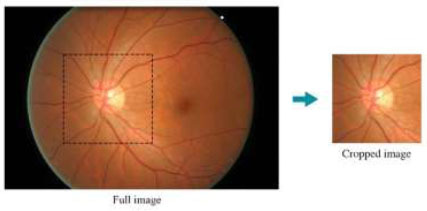
Comparison between full and cropped RFIs from the ORIGA dataset.

**Fig. (2):**
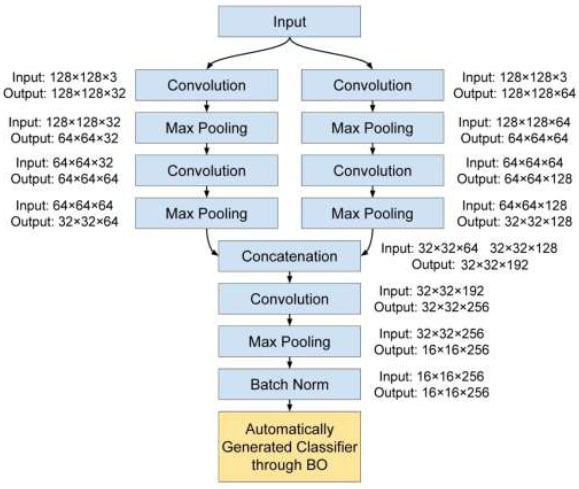
Structure of the proposed feature extraction module within the multi-branch CNN. Conv: convolution; Maxpool: Max pooling; Batch Norm: Batch normalization.

**Fig. (3):**
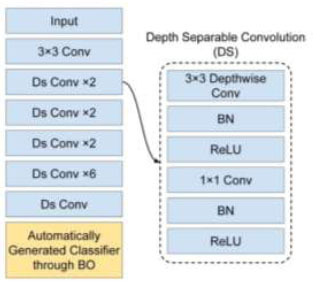
Customized mobilenet architecture.

**Fig. (4):**
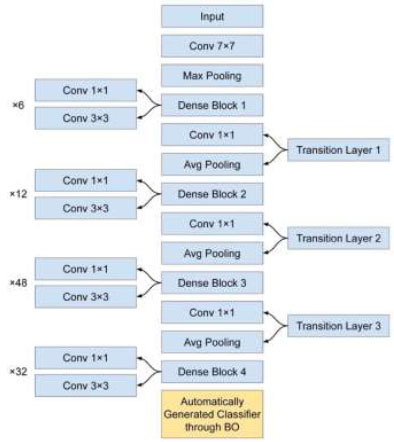
Customized densenet201 architecture.

**Fig. (5):**
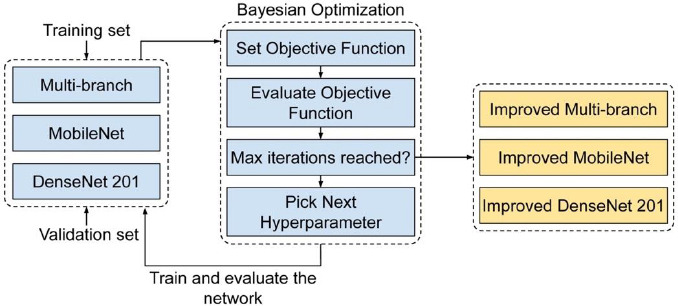
Proposed hyperparameters optimization flow.

**Fig. (6):**
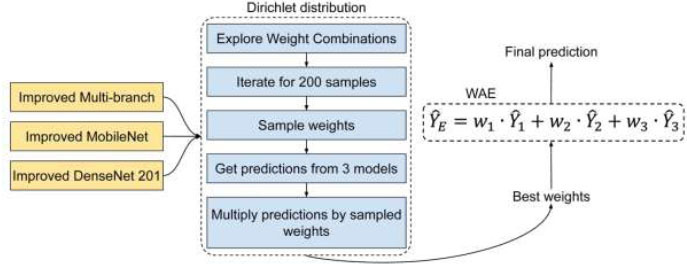
Flow of our proposed dirichlet-based WAE.

**Fig. (7):**
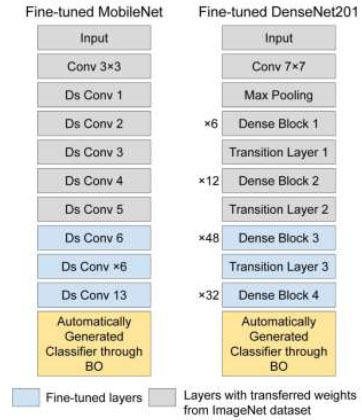
Fine-tuned MobileNet and DenseNet201 architectures.

**Fig. (8):**
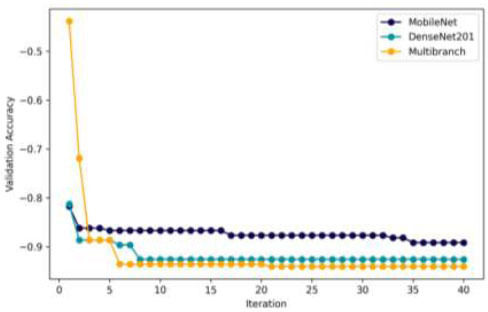
Progress of BO for our deep models.

**Fig. (9):**
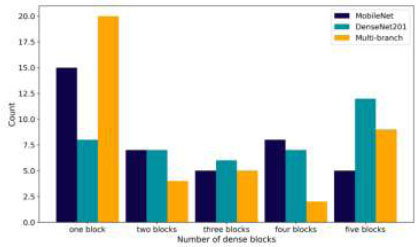
Number of dense block selections for our CNN Models.

**Fig. (10):**
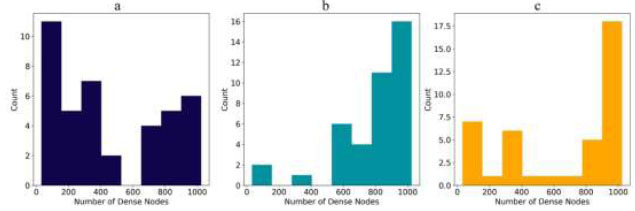
Number of dense node selections for our CNN Models. (**a**) MobileNet; (**b**) DenseNet201; (**c**) Multi-branch.

**Fig. (11):**
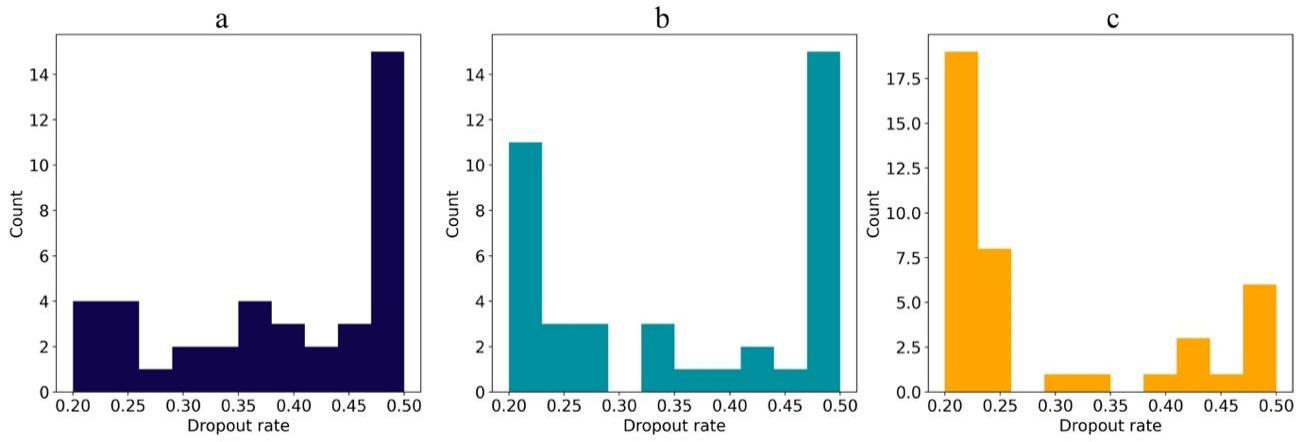
Dropout rate selections for our CNN Models. (**a**) MobileNet; (**b**) DenseNet201; (**c**) Multi-branch.

**Fig. (12):**
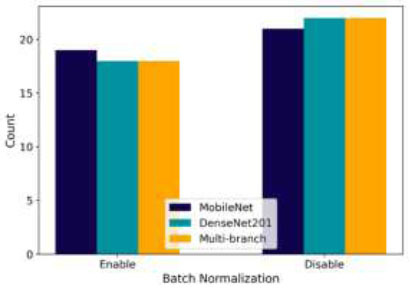
BN preferences across our CNN Models.

**Fig. (13):**
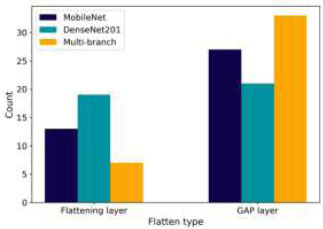
Distribution of flatten type preferences among our CNN Models.

**Fig. (14):**
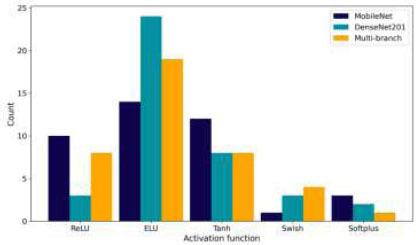
Count of activation function preferences across our CNN Models.

**Fig. (15):**
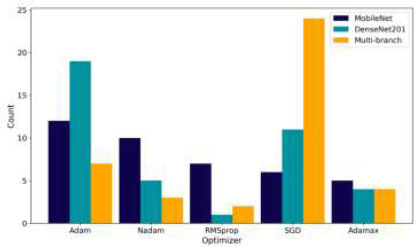
Comparison of optimizer preferences in our CNN models.

**Fig. (20):**
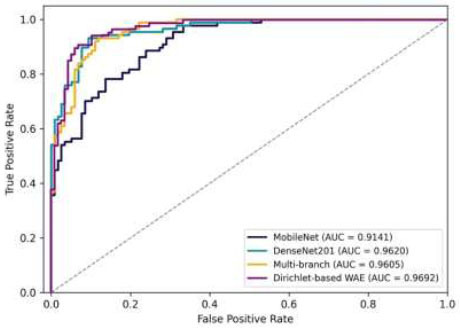
Comparison of the ROC curves for the MobileNet, DenseNet201, Multi-branch, and ensemble models on the test set.

**Table 1:** Hyperparameters setting.

Hyperparameter	Search Dimension
Number of dense blocks	1 to 5
Number of dense nodes	32 to 1024
Dropout rate	0.2 to 0.5
BN	Enabled, Disabled
Flattening Type	Flattening Layer, GAP Layer
Activation Function	ReLU, ELU, Tanh, Swish, Softplus
Optimizer	Nadam, RMSProp, Adam, SGD, AdaMax

**Table 2:** Impact of hyperparameter tuning on our GD models.

Model	Dense Blocks	Dense Nodes	Dropout Rate	BN	Flatten Type	Activation Function	Optimizer	ACC
Pre-tuned MobileNet	1	362	0.435	Yes	Flatten	Softplus	Nadam	81.77
Pre-tuned DenseNet201	3	351	0.234	Yes	GAP	ReLU	Nadam	81.28
Pre-tuned Multi-branch	3	351	0.234	Yes	GAP	ReLU	Nadam	43.84
Post-tuned MobileNet	1	32	0.5	Yes	Flatten	ELU	RMSProp	89.16
Post-tuned DenseNet201	5	797	0.207	No	Flatten	ReLU	Adamax	92.61
Post-tuned Multi-branch	3	821	0.2	No	Flatten	ReLU	SGD	94.08

**Table 3:** Performance evaluation metrics for the proposed models on the validation set.

Model	Label	PREC	SEN	F1	ACC	AUC
Improved MobileNet	Normal	92.79	88.03	90.35	89.16	0.9425
	Glaucoma	84.78	90.7	87.64		
	Average	88.79	89.37	89		
Improved DenseNet201	Normal	92.5	94.87	93.67	92.61	0.9548
	Glaucoma	92.77	89.53	91.12		
	Average	92.64	92.2	92.40		
Improved Multi-branch	Normal	92.68	97.44	95	94.08	0.9829
	Glaucoma	96.25	89.53	92.77		
	Average	94.47	93.49	93.89		
Dirichlet-based WAE	Normal	95	97.44	96.2	95.56	0.9877
	Glaucoma	96.39	93.02	94.67		
	Average	95.69	95.23	95.44		

**Table 4:** Performance evaluation metrics for the proposed models on the test set.

Model	Label	PREC	SEN	F1	ACC	AUC
Improved MobileNet	Normal	83.48	82.05	82.76	80.39	0.9141
	Glaucoma	76.40	78.16	77.27		
	Average	79.94	80.11	80.02		
Improved DenseNet201	Normal	92.17	90.60	91.38	90.19	0.962
	Glaucoma	87.64	89.66	88.64		
	Average	89.91	90.13	90.01		
Improved Multi-branch	Normal	89.83	90.60	90.21	88.72	0.9605
	Glaucoma	87.21	86.21	86.71		
	Average	88.52	88.40	88.46		
Dirichlet-based WAE	Normal	93.10	92.31	92.70	91.66	0.9692
	Glaucoma	89.77	90.80	90.29		
	Average	91.44	91.56	91.49		

**Table 5:** Performance comparison of optimized models with and without fine-tuning after 16 and 32 epochs.

Model	Epochs	Fine-tuning	Dataset	PREC	SEN	F1	ACC	AUC
Improved MobileNet	16	No	Validation	88.79	89.37	89	89.16	0.9425
	32	Yes		96.41	96.55	96.47	96.55	0.9942
	16	No	Test	79.94	80.11	80.02	80.39	0.9141
	32	Yes		94.89	94.12	94.44	94.60	0.9855
Improved DenseNet201	16	No	Validation	92.64	92.2	92.40	92.61	0.9548
	32	Yes		96.30	95.66	95.94	96.05	0.9919
	16	No	Test	89.91	90.13	90.01	90.19	0.9620
	32	Yes		95.02	93.25	93.88	94.11	0.9754
Improved Multi-branch	16	No	Validation	94.47	93.49	93.89	94.08	0.9829
	32	No		95.97	95.97	95.97	96.05	0.9832
	16	No	Test	88.52	88.40	88.46	88.72	0.9605
	32	No		90.65	90.26	90.44	90.68	0.9613
Dirichlet-based WAE	16	-	Validation	95.69	95.23	95.44	95.56	0.9877
	32	-		**96.92**	**96.08**	**96.44**	**97.04**	**0.9971**
	16	-	Test	91.44	91.56	91.49	91.66	0.9692
	32	-		**95.51**	**94.55**	**94.94**	**95.09**	**0.9854**

**Table 6:** Comparison with existing approaches in automated GD.

Reference	Method	Dataset	# of images	ACC (%)	AUC
Maheshwari *et al.* [19] (2017)	VMD for decomposition, Entropy and Fractal features, Relief for feature selection, and LS-SVM for classification	Local dataset	488	0.951	-
Agrawal *et al.* [17] (2019)	QB-VMD and SVM	RIM-ONE	505	86.1	-
Kirar *et al.* [18] (2019)	(DWT + EWT + DWTEWT + EWTDW) for feature extraction and SVM for classification	RIM-ONE	505	83.6	-
			150	88.7	-
Bajwa *et al.* [20] (2019)	RCNN for ROI extraction and 7-layer CNN for classification	ORIGA	650	-	0.874
Liao *et al.* [23] (2020)	EAMNet	ORIGA	650	-	0.88
Martins *et al.* [26] (2020)	MobileNetV2	Merged 7 datasets: ORIGA, Drishti-GS, RIM-ONE r1, RIM- ONE r2, RIM-ONE r3, iChallenge, and RIGA	3,231	87	0.93
Serte *et al.* [28] (2021)	AlexNet+ResNet-50+ResNet-152	Harvard dataset	1,542	88	0.94
	AlexNet+ResNet-50			88	0.95
Cho *et al.* [27] (2021)	Ensemble of 56 CNNs	Local dataset	3,460	88.1	0.975
Almansour *et al.* [25] (2022)	R-CNN for ROI extraction and VGG16 for classification	Merged 7 datasets: RIGA (Bin Rushed), RIGA (Magrabi), HRF, Kaggle, ORIGA, Eyepacs, and KAIMRC	3,771	78	0.87
Leonardo *et al.* [24] (2022)	EfficientNetB0	Merged 7 datasets: ORIGA, Drishti-GS, REFUGE, RIM-ONE r1, RIM-ONE r2, RIM- ONE r3, and ACRIMA	3,187	93.1	-
Juneja *et al.* [22] (2022)	CoG-NET	Merged 4 datasets: Drishti-GS, RIM-ONE, REFUGE, and ACRIMA	2,172	93.5	-
D'Souza *et al.* [21] (2024)	AlterNet-K	Rotterdam EyePACS AIROGS	113,893	91.6	0.968
**Proposed Method**	Improved MobileNet	Merged 2 datasets: ORIGA and ACRIMA	1,187	94.6	0.985
	Improved DenseNet201			94.1	0.975
	Improved Multi-branch			90.6	0.961
	**Dirichlet-based WAE**			**95**	**0.985**

## Data Availability

The data supporting the findings of this study is publicly available on the Figshare platform: (ACRIMA: https://figshare.com/s/c2d31f850af14c5b5232) and (ORIGA: https://figshare.com/articles/dataset/Retinal_Fun dus_Glaucoma_Image_dataset/24549217?file=43119880).

## LIST OF ABBREVIATIONS

GD: Glaucoma Detection
CNN: Convolutional Neural Network
BO: Bayesian Optimization
EL: Ensemble Learning
OD: Optic Disc
ML: Machine Learning
AI: Artificial Intelligence
SVD: Singular Value Decomposition
DWT: Discrete Wavelet Transform
EWT: Empirical Wavelet Transform
SVD: Singular value decomposition
VMD: Variational Mode Decomposition
LS-SVM: Least Squares Support Vector Machine
BN: Batch Normalization
EAM: Evidence Activation Mapping
TL: Transfer Learning
ROI: Region-of-interest
GAP: Global Average Pooling
